# Comparison of refraction, presbyopia, and dry eye-related parameters between monofocal and multifocal contact lens users

**DOI:** 10.1007/s10384-026-01384-7

**Published:** 2026-06-04

**Authors:** Masahiko Ayaki, Akiko Hanyuda, Kazuno Negishi

**Affiliations:** Department of Ophthalmology, Keio University School of Medicine, 35 Shinanomachi, Shinjuku-ku, Tokyo, 160-8582 Japan

**Keywords:** Presbyopia, Contact lens, Multifocal lens

## Abstract

**Purpose:**

To investigate factors associated with multifocal contact lens (CL) use or prescription for presbyopia correction.

**Study design:**

Retrospective cross-sectional

**Methods:**

Overall, 3264 CL users were assessed and categorized into three age groups; 40s (aged 40–49 years), 50s (aged 50–59 years), and 60s (aged 60–69 years). Refraction, near add power, dry eye-related parameters, and visual acuity were compared between monofocal and multifocal CL users in each age group. The odds ratios of factors for using multifocal CLs were calculated.

**Results:**

Multifocal CL users tended to be older across all age groups and were more likely to be male, aged in their 40s and 50s, and have lower degrees of myopia and astigmatism. In the 50s and 60s groups, multifocal CL users also showed better CL-corrected visual acuity, smaller differences between best-corrected and CL-corrected vision, and less under-correction. Near addition of CL was significantly higher in multifocal CL users in the 40s group. Among dry eye-related parameters, multifocal CL users had higher Schirmer values in the 40s and 60s groups, longer tear break-up time in the 50s group, and fewer keratopathies in the 40s and 50s groups. Odds ratios for multifocal CL use were significant for age, spherical equivalent, and astigmatism across all age groups, Schirmer values in the 40s and 50s groups, and tear break-up time and keratopathy were significantly higher in the 50s group.

**Conclusion:**

Multifocal CLs were more likely to be prescribed for users with lower refractive errors and healthier ocular surfaces. The current findings may contribute to optimizing CL prescriptions and presbyopia management strategies.

## Introduction

Presbyopia is an age-related progressive disorder of the accommodation system, primarily characterized by a gradual loss of the elasticity of the crystalline lens and its ability to dynamically adjust focus for near objects [1–3]. The recent increase in the reliance on digital interfaces has amplified the functional burden on near vision, and presbyopia is now, not only a clinical concern, but also a lifestyle challenge [4, 5]. Indeed, presbyopia is emerging as a significant public health and societal issue leading to reduced productivity [6] and decline in quality-of-life [7]. Moreover, the economic consequences of presbyopia are considerable, encompassing direct costs, such as corrective techniques and medical expenses [8].

Correction of presbyopia [9, 10] depends on visual needs and lifestyle, and includes reading eyeglasses, progressive/bifocal eyeglasses, monofocal and multifocal contact lenses (CLs), eyeglasses with CLs, topical medication [11], and surgical options as well as monovision technique. Of these, eyeglasses and CLs are the most popular and easily prescribed at vision care clinics. A 2023 survey of the Japanese population [12] indicates that the proportion of CL users was 18.3% of those aged 20 to 79 (n = 9289), although the results for aged users was not available. A national Korean survey [13] in 2022 states that the main changes were an increase in middle-aged CL users compared to the early 2000s.

Multifocal CLs are typically made from hydrogel or silicone hydrogel polymers and designed to provide simultaneous vision correction at multiple distances. The advantages of multifocal CLs are convenience without carrying reading glasses or switching between different pairs of glasses. Multifocal CLs allow smoother transitions between focal points, avoiding the musculoskeletal strain due to the required head movement to find the right focal zone when using progressive eyeglasses. Disadvantages of multifocal CLs include the adaptation period for adjustment to the layered prescriptions and learning to focus correctly, visual compromise for reduced contrast sensitivity or ghosting [14], and cost. There is a gap between Japan’s growing presbyopic population, reported as 29.3% of those aged over 64 years [15], and the underutilization of multifocal CLs since the prescription of multifocal CLs is far less than that of monofocal CLs [16]. Age-related visual deterioration may lead to difficulty in adapting to multifocal CLs. For example, decreased accommodation amplitude, reduced contrast sensitivity, increased glare sensitivity, and diminished pupillary reaction could be barriers to using multifocal CLs [17–19]. Dry eyes and decreased macular sensitivity, and slow adaptation, may also be factors against using multifocal CLs. However, there is little information about multifocal CL users [20–22]. The aim of this study was to characterize the visual acuity and refraction of patients using multifocal CLs compared with monofocal CL users to explore relevant candidates for multifocal CLs for presbyopic correction to better optimize CL prescription for patients with presbyopia.

## Methods

### Study design and participants

This retrospective, clinic-based study involved consecutive healthy individuals who visited the Otake Eye Clinic in Kanagawa, Japan, for routine care. The study protocol received approval from the Institutional Review Board and Ethics Committee of the Kanagawa Medical Association (approval date: November 12, 2018; approval number: krec2059006) and adhered to the principles outlined in the Declaration of Helsinki. As the study employed an opt-out approach, the requirement for informed consent was waived by the same ethics committee.

Data were collected through a retrospective chart review of consecutive patients who attended the clinic between December 2018 and October 2024. Additionally, the Institutional Review Board and Ethics Committee of Keio University School of Medicine granted approval (approval date: May 31, 2024; approval number: 20241019) to allow authorship for investigators (KN, AH, and MA) affiliated with Keio University. The study protocol was registered with the UMIN Clinical Trials Registry (UMIN000051891) on August 15, 2023. All data analyzed in this study were obtained as part of routine standard-of-care ophthalmic examinations.

### Inclusion and exclusion criteria

Eligible participants were CL users aged 40 to 69 years, with bilateral phakic eyes and best-corrected visual acuity of 20/30 or better. Included CLs were daily disposable soft CLs of either monofocal or multifocal (bifocal) type. Exclusion criteria comprised any history of corneal or intraocular surgery, including laser procedures, refractive surgery, or cataract extraction. Patients with moderate to severe cataracts (defined as nuclear cataract grade ≥2 per WHO grading), glaucoma, macular pathology, or severe dry eye disease, characterized by both symptomatic complaints and dense central keratoconjunctival staining, were excluded.

### Ophthalmological examinations

Participants underwent comprehensive ophthalmic evaluation, including best-corrected visual acuity testing (Vision Chart SSC-370R; Nidek Co., Ltd.), autorefractometry, intraocular pressure measurement (Tonoref™ II; Nidek Co., Ltd.), slit-lamp biomicroscopy, fundus examination, and standard automated perimetry using the Humphrey Visual Field Analyzer with the Swedish Interactive Threshold Algorithm Standard 24-2 program (Carl Zeiss Meditec).

Binocular near add power was assessed at a distance of 30 cm using a Bankoku near-acuity chart (Handaya Inc.). After determining the distance refractive correction, the minimum additional lens power required to achieve near visual acuity better than 20/25 at 30 cm was measured in 0.25 D increments and recorded as the near add power.

Dry eye assessments included tear break-up time (BUT), Schirmer test, and corneal staining. BUT was measured using fluorescein-impregnated filter paper strips (Ayumi Pharmaceutical) and defined as the interval between the third blink and the appearance of the first dry spot on the corneal surface. The mean of three measurements was used. Corneal staining was evaluated one minute after fluorescein dye instillation.

### Statistical analysis

Patients were classified into three age groups: the 40s group (aged 40 to 49 years), the 50s group (aged 50 to 59 years), and the 60s group (aged 60 to 69 years). Patient demographics and ophthalmological parameters are presented as mean ± standard deviation for continuous variables and as percentages for categorical variables for each age group.

Parameters between monofocal and multifocal CL users were compared among these age groups using t-tests or chi-squared tests, as appropriate. Consequently, the following explanatory variables were selected: age, sex, spherical equivalent, astigmatic errors, anisometropia, near add power, Schirmer test value, tear break-up time, and positive corneal staining (superficial punctate keratopathy; SPK). The odds ratios and 95% confidence intervals for using multifocal CLs in relation to each selected ophthalmic parameter was then estimated using logistic regression models. Each variable was analyzed in a separate model adjusted for age and sex. All analyses were performed using StatFlex (Atech), with a two-sided P-value < 0.05 considered to indicate a significant difference.

## Results

**Fig. 1:**
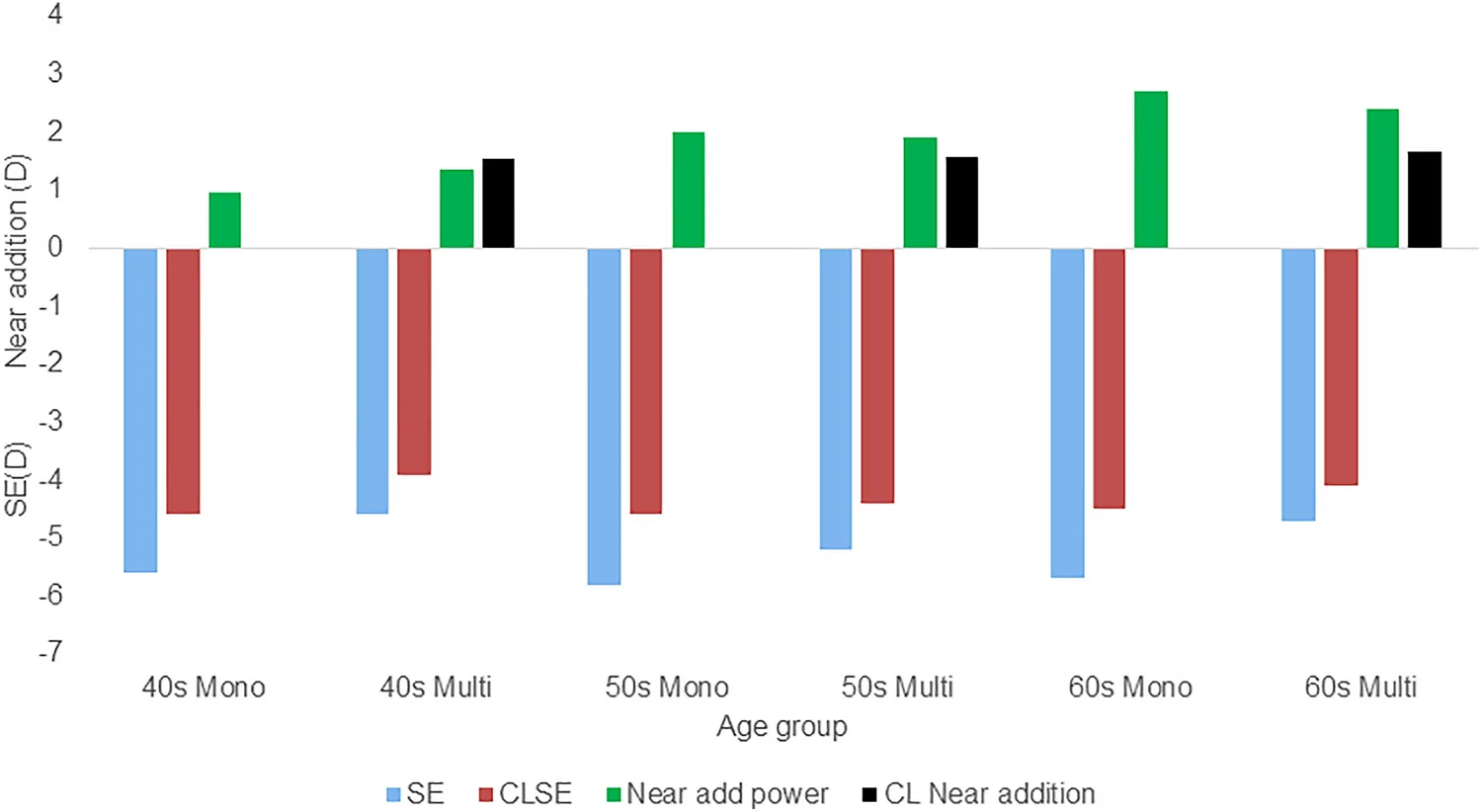
Spherical equivalent (SE) and near add power of monofocal (Mono) and multifocal (Multi) contact lens (CL) users. There was undercorrection of SE in all groups, and its magnitude was larger in the monofocal than the multifocal group. Near add power with eyeglasses was age-dependent among multifocal CL users (P < 0.01), whilst near addition of multifocal CLs was similar across all age groups (P = 0.08). Near addition of multifocal CLs was smaller than near add power with eyeglasses in the 50s (P < 0.01) and 60s (P < 0.01) age groups, while it was larger in the 40s age group (P < 0.05)

Patient demographics and ophthalmological parameters, and the number of participants in each age group, are shown in Table 1. The proportion of multifocal CL users was 15.5% (458 of 2959) in the 50s and 60s age groups. The results of the comparison of parameters between monofocal and multifocal CL users are: regarding refraction, multifocal CL users were less myopic (all groups; Fig. 1) and less astigmatic (all groups) compared with monofocal CL users. Regarding presbyopic parameters, multifocal CL users had more near add power (40s group) and less near add power (50s and 60s groups) compared with monofocal CL users (Fig. 1). Addition of multifocal CLs was similar among all age groups (40s, 50s, and 60s; P = 0.33). Regarding habitual vision and correction with CLs, multifocal CL users preferred better CL-corrected vision (50s and 60s groups), less difference between best corrected vision and CL-corrected vision (50s and 60s groups), less astigmatic correction (all groups) in CLs, and less under-corrected refraction in CLs (50s and 60s groups) compared with monofocal CL users. Regarding dry eye-related parameters, multifocal CL users had greater Schirmer test values (40s and 60s groups), longer BUT (50s group) and less SPK (40s and 50s groups) compared with monofocal CL users. Regarding retinal thickness, multifocal CL users had thicker RNFLs (40s and 50s groups). The analysis of presbyopic parameters in multifocal CL users (Fig. 1) indicated that near add power with eyeglasses was age-dependent in the multifocal CL users (P < 0.01), whilst near addition of multifocal CLs was similar across all age groups (P = 0.08). Near addition of multifocal CLs was smaller than near add power with eyeglasses in the 50s (P < 0.01) and 60s (P < 0.01) groups, while it was larger in the 40s group (P < 0.05).

**Table 1:** Patient demographics and ophthalmological parameters

	40s (n = 1505)		50s (n = 2410)		60s (n = 549)	
Parameters	Monofocal (n = 1379)	Multifocal (n = 126)	Monofocal (n = 1997)	Multifocal (n = 413)	Monofocal (n = 317)	Multifocal (n = 232)
Age, y	44.5 (2.8)	46.5 (2.2)**	53.5(2.7)	54.3 (2.8)**	63.6 (2.5)	63.1 (2.6)*
% Men	23.4	34.1**	31.6	49.6**	61.5	68.1
% Multifocal CL		3.1		9.8		40.4
Spherical equivalent, D	−5.63 (2.82)	−4.66 (2.50)**	−5.84 (3.15)	−5.25 (2.89)**	−5.75 (3.98)	−4.76 (3.26)**
Astigmatic errors, D	0.55 (0.88)	0.38 (0.62)*	0.56 (0.88)	0.27 (0.50)**	0.61 (0.75)	0.49 (0.73)
Anisometropia, D	0.65(0.82)	0.71 (0.64)	0.77 (1.01)	0.71 (0.84)	1.00 (1.32)	0.80 (1.24)
Near add power, D	0.97 (0.81)	1.34 (0.67)*	2.06 (0.56)	1.96 (0.68)	2.77 (0.43)*	2.48 (0.73)
Difference of refraction and corrected visual acuity between glasses and CL						
SE of CL, D	−4.66 (2.36)	−3.97 (2.13)*	−4.69 (2.58)	−4.47 (2.44)	−4.52 (3.04)	−4.10 (2.77)
Δ SE, D	0.41 (0.66)	0.36 (0.57)	0.50 (0.82)	0.30 (0.58)**	0.59 (0.77)	0.27 (0.72)**
Astigmatic errors of CL, D	0.13 (0.41)	0.03 (0.18)**	0.22 (0.51)	0.05 (0.25)**	0.14 (0.42)	0.01 (0.09)**
Δ Astigmatic errors, D	0.30 (0.67)	0.24 (0.47)	0.22 (0.61)	0.17 (0.48)	0.33 (0.57)	0.38 (0.64)
Anisometropia of CL, LogMAR	0.01 (0.04)	0.01 (0.02)	0.02 (0.06)	0.01 (0.04)*	0.02 (0.05)	0.03 (0.06)
Anisometropia of CL, D	0.50 (0.78)	0.61 (0.63)	0.57 (0.77)	0.60 (0.67)	0.79 (1.10)	0.68 (0.97)
VA with CL, LogMAR	−0.02 (0.08)	−0.02 (0.06)	−0.05 (0.12)	−0.02 (0.07)	−0.06 (0.11)	−0.03 (0.09)*
Δ VA	0.09 (0.08)	0.10 (0.06)	0.13 (0.12)	0.10 (0.07)**	0.14 (0.11)	0.11 (0.09)*
Near add power of CL, D		1.54 (0.51)		1.56 (0.52)		1.65 (0.52)
Δ Near add power, D		−1.24 (0.77)		−1.05 (1.03)		−1.05 (1.22)
Schirmer test value, mm	14.7 (8.7)	19.1 (9.3)*	14.1 (8.3)	14.9 (7.6)	10.5 (7.1)	16.4 (7.3)**
Tear break-up time, s	3.5 (2.3)	3.6 (2.9)	3.1 (2.3)	3.7 (2.2)*	3.8 (2.4)	3.4 (1.8)
SPK (yes/no), %	22.5	16.8	23.3	11.8*	8.9	7.0

Values are mean (standard deviation) unless otherwise indicated. Δ = value with CL—value with eyeglasses, *P < 0.05, **P < 0.01, vs. monofocal group, unpaired t test or chi-squared test, as appropriate

*CL* contact lenses, *SE* spherical equivalent, *VA* visual acuity, *SPK* superficial punctate keratopathy

Logistic regression analysis demonstrated that age, male sex, spherical equivalent, and astigmatic errors were strong factors for using multifocal CLs across all age groups (Table 2). The Schirmer test value, tear break-up time, and SPK were also significant in multiple age groups.

**Table 2:** Comparison of odds ratios of factors for using multifocal contact lenses

	Age group		
Characteristics	40s	50s	60s
Age (y)	1.34 (1.24–1.45)**	1.11 (1.06–1.16)**	0.91 (0.89–0.98)*
Sex (man = 1)	1.64 (1.10–2.45)*	1.98 (1.54–2.54)**	1.28 (0.89–1.83)
Spherical equivalent (D)	1.01 (1.00–1.01)**	1.01 (1.00–1.01)**	1.01 (1.00–1.01)**
Magnitude of astigmatic errors (D)	0.99 (0.99–0.99)**	0.99 (0.99–0.99)**	0.99 (0.99–1.00)**
Magnitude of anisometropia (D)	1.00 (0.99–1.00)	0.99 (0.99–1.00)	0.99 (0.99–1.00)
Near add power (D)	1.11 (0.63–1.95)	0.69 (0.48–0.99)*	0.47 (0.26–0.83)*
Schirmer test (mm)	1.06 (1.00–1.11)*	1.01 (0.98–1.05)	1.12 (1.03–1.22)**
Tear break-up time (s)	1.09 (0.95–1.24)	1.11 (1.02–1.21)*	0.83 (0.69–0.99)*
SPK (yes/no)	0.73 (0.41–1.28)	0.54 (0.34–0.80)**	0.79 (0.40–1.57)

Each variable was analyzed in a separate model adjusted for age and sex. Odds ratio (95% confidence interval). *P < 0.05, **P < 0.01

*SPK* superficial punctate keratopathy

## Discussion

Multifocal CL users were older across all age groups, consistent with the presbyopia progression. Male predominance was observed among multifocal CL users in the 40s and 50s groups, the reasons for which remain unknown. A Japanese survey indicates a myopic shift and faster myopic progression in adolescent women [23] and suggests that women tended to view near objects throughout life. Women may prefer to correct near vision with eyeglasses rather than multifocal CLs since visual quality is better with eyeglasses than multifocal CLs, especially for the elderly whose visual function tends to be lower [17–19].

The amount of near addition in multifocal CLs was similar among the age groups; this is paradoxical since near add power is distinctly age-dependent. These findings may indicate that the add power of multifocal CLs tends to converge within a limited range in actual clinical practice, possibly because of fitting constraints and the trade-off with distance visual performance. Overall, the characteristics of multifocal CL users were not uniform across age groups since near add power was higher in multifocal CL users in the 40s group, but lower in the 50s and 60s groups, and dry eye-related findings also differed by age group. Therefore, individualized work-up based on near add power and ocular surface may be crucial for prescription of multifocal CLs.

Despite both study groups being CL users, the significant differences were longer tear BUT and less SPK in multifocal CL users, whereas Schirmer values were not significantly different in the 50s group. Differences were observed in tear film stability and SPK rather than in tear secretion itself. A previous investigation suggests that women in this age group had prevalent ocular surface and visual problems [17], possibly leading to difficulty in adopting multifocal CLs. The adaptation to multifocal CLs may be harder for this age group due to their negative factors.

Multifocal CL users had healthier ocular surfaces and less refractive errors than monofocal CL users, and did not prefer to compromise far vision by using multifocal CLs. These results may reflect that individuals with healthier ocular surfaces and lower refractive errors are more likely to be prescribed, or successfully continue wearing, multifocal CLs, and that they could adapt to multifocal CLs more smoothly than other patients.

Several factors may contribute to the gap between the growing presbyopic population and the underutilization of multifocal CLs in Japan. It is a cultural characteristic that, unlike other developed countries that use alphabet-based languages, Japanese often prioritize visual clarity; multifocal CLs can introduce visual compromises, including halos or reduced contrast [14]. Additionally, older adults tend to prefer eyeglasses for reading and close work. Many younger users favor CLs for cosmetic reasons, especially among women; however, multifocal CLs are typically prescribed for older adults who may be less similarly influenced, and fine and close make-up may be difficult with multifocal CLs. Maintaining personal appearance and grooming behavior are closely linked to psychological well-being, self-esteem, and social participation among older women [24, 25]. Additionally, multifocal CLs require neuroadaptation [26], and some users experience blurry vision during the adjustment period which discourages adoption. Dry eye is also a common problem among the elderly and a barrier to using multifocal CLs. Furthermore, vision care professionals may be hesitant to recommend multifocal CLs due to the time-intensive fitting process and adaptation challenges [27]. Despite the clinical needs, such barriers may outweigh the benefits.

The strengths of this study include the comprehensive investigation of ophthalmological parameters and comparison between monofocal and multifocal CL users. The current results should provide useful insights for presbyopia correction with CLs in a super-aging society where visual needs and demand for relevant strategies to address presbyopia are rapidly growing.

This study has several limitations. It has a potential prescription or adaptation bias. As this is a clinic-based retrospective analysis, the multifocal group may represent patients who were considered suitable candidates for multifocal lenses or who successfully adapted to them. Patients who did not tolerate multifocal lenses or were not offered any may not have been recruited. Preference of CLs and presbyopic correction depends on lifestyle, hobbies, occupation, working distance, and other potential factors. Further research with investigation of these factors is warranted to seek and provide optimal and personalized presbyopia correction. Additionally, multicenter studies with cultural and industrial diversity are warranted to extend the present results. The current study includes CL users only and does not represent preferred presbyopia correction in the whole population.

In conclusion, presbyopia correction with multifocal CLs was more often prescribed for CL users with relatively healthier ocular surfaces and less refractive errors. The current results emphasize the importance of individualized CL prescriptions based on age-specific visual demands and ocular health, contributing to more effective presbyopia management strategies.
